# The fusion subunit vaccine L-DBF protects aged mice against heterologous lethal *Shigella* challenge after prior exposure

**DOI:** 10.3389/fimmu.2025.1586537

**Published:** 2025-06-05

**Authors:** Md Shafiullah Parvej, Ti Lu, Suhrid Maiti, Zackary K. Dietz, Debaki R. Howlader, Mst Nusrat Zahan, Alexa Cato, Satabdi Biswas, William D. Picking, Wendy L. Picking

**Affiliations:** Bond Life Sciences Center and the Department of Veterinary Pathobiology, University of Missouri, Columbia, MO, United States

**Keywords:** *Shigella*, subunit vaccine, aged mice, intranasal vaccination, pre-exposure

## Abstract

Shigellosis is among the top causes of bacterial diarrhea with significantly high morbidity and mortality for children under five years of age in low- and middle-income countries. Unfortunately, there are currently no licensed vaccines to prevent shigellosis. Our laboratory has developed the protein-based subunit vaccine candidate L-DBF by fusing the type III secretion system proteins IpaB and IpaD with the mucosal adjuvant LTA1, the active moiety of the heat-labile toxin from enterotoxigenic *Escherichia coli*. L-DBF delivered intranasally has been shown to protect against multiple serotypes of *Shigella* using the lethal pulmonary mice model. In recent work, we showed that even following prior infection with two sublethal doses of *S. flexneri* 2a in adult mice, L-DBF effectively cross-protects mice from a potentially lethal challenge with *S. sonnei* in addition to *S. flexneri*. We therefore wanted to know whether a prior sublethal infection by *S. flexneri* or *S. sonnei* would impact the protective immune response elicited by L-DBF against heterologous lethal infections in an aged mouse model. Elderly mice were subjected to a single sublethal dose of *S. flexneri* or *S. sonnei* prior to vaccination with L-DBF. We found that pre-exposed mice receiving L-DBF developed heterologous cross-protection against both *S. flexneri* and *S. sonnei* lethal challenge doses. This contrasts with unvaccinated mice that succumbed to the lethal challenge, despite a single homologous or heterologous pre-exposure to *Shigella* spp. Thus, L-DBF is a strong candidate for a protective shigellosis vaccine in all age groups to protect against commonly encountered *Shigella* serotypes in both naïve hosts and those pre-exposed with homologous or heterologous *Shigella* serotypes.

## Introduction

Shigellosis, also known as bacillary dysentery, is caused by the bacteria in the genus *Shigella*. Bacillary dysentery is one of the leading causes of diarrheal deaths accounting for 212,438 deaths globally in 2016 alone (1, 2). Among the shigellae, *S. flexneri* and *S. sonnei* account for 90% of shigellosis cases worldwide (3). *S. flexneri* is the dominant cause of shigellosis in low socioeconomic countries (4), while *S. sonnei* is more prevalent in industrialized regions (5). Recent epidemiological studies, however, report that *S. sonnei* is replacing *S. flexneri* as the dominant cause of shigellosis in developing countries (6, 7). This might be due to the ability of *S. sonnei* to acquire antibiotic-resistance genes from other commensals and pathogens (7). Additionally, *S. sonnei* has an increased ability to grow in *Acanthamoeba* in public water supplies, which acts as a surrogate host that allows it to persist in adverse environmental conditions (6, 8). Thus, prevention of both groups is essential to reduce shigellosis cases and associated deaths globally. Antibiotic therapy is the only available treatment option for bacterial diarrhea and shigellosis worldwide, however, emerging antibiotic resistance makes it challenging to treat multi-drug resistance (MDR) bacterial infections. The World Health Organization (WHO) listed *Shigella* spp as a priority pathogen for MDR (9). Thus, the emerging antibiotic resistance of *Shigella* spp worldwide will make it more difficult to treat shigellosis (2, 10, 11). A protective vaccine against all *Shigella* serotypes would be the most effective method to control shigellosis.

*Shigella* spp use a type-III secretion system apparatus (T3SA) to inject virulence effector proteins into host cells to promote invasion and trigger the onset of infection (12). The T3SA tip protein IpaD and effector protein IpaB are immunogenic and highly conserved in all *Shigella* serotypes (13). Thus, unlike LPS-based vaccines, a vaccine candidate including IpaB and IpaD would be effective in preventing infection by multiple *Shigella* serotypes. We showed that intranasal (IN) administration of IpaB and IpaD admixed with the mucosal adjuvant double-mutant heat-labile toxin (dmLT) from enterotoxigenic *Escherichia coli* (ETEC) protected mice against a lethal pulmonary model (13). Likewise, a genetic fusion of IpaD and IpaB (DBF) admixed with dmLT induced a similar protective immune response against a pulmonary lethal mouse model (14). To further lower production and development costs, LTA1, the active moiety of LT, was genetically fused to the N-terminus of DBF to produce a single self-adjuvanting protein, L-DBF. LTA1 alone, delivered IN, does not induce the toxic effects (e.g. Bell’s palsy) that can be associated with dmLT (15). L-DBF induced similar IpaB and IpaD immune responses in the mouse model and protected these mice from lethal pulmonary challenge following IN immunization (16). Since the worldwide prevalence of infections by MDR *Shigella* spp is occurring in populations residing in developing countries, there is the potential for spread globally by international travel (17). Thus, an effective vaccine in needed to induce protective immunity among individuals who have a prior history of shigellosis. Previously, we tested L-DBF in mice pre-exposed to two sublethal doses of *S. flexneri* before vaccination and reported that L-DBF protected mice in a pulmonary lethal challenge by *S. flexneri* without any adverse effects on existing immunity in adult mouse models (18).

While shigellosis generally occurs in all age groups, children younger than five years old and elderly people over 70 years old are highly susceptible to having poor outcomes (19). The most effective preventive measure for reducing infections and newborn mortality worldwide would be vaccination at an early age. However, older populations may not be as effectively protected by the vaccine systems currently used globally. Though elderly people are more prone to vaccine-preventable diseases, most of them remain unvaccinated in the United States (20). Since the world’s population is aging at a rapid rate, protecting the elderly from infectious diseases is an unmet public health need. According to United Nations estimates, nearly 25% of the world’s population will be over 65 by 2050, with 75% of these elderly people residing in LMICs (21). Because the immune system of the elderly undergoes immunosenescence, existing vaccines are often less immunogenic and, hence, less effective in this population (22). Thus, new vaccinations that are particularly adapted to boosting protective immunity in an aging population are needed (23). The immune response alterations observed in the elderly can be attributed to intrinsic flaws in immune cells, which exhibit modified phenotype and function, as well as potential defects in the lymphoid organs including bone marrow and thymus (24–26). Aging has a detrimental effect on bone marrow’s ability to produce B cells, which lowers the quantity of B cells (27) and as people age their antibody diversity and affinity decreases, resulting in compromised antibody responses (27). Furthermore, as impaired innate and adaptive immune responses make the elderly more vulnerable to pathogens and decreases the effectiveness of many vaccines (28).

In this investigation, elderly mice pre-exposed once to either *S. flexneri* or *S. sonnei* were vaccinated with formulated L-DBF to assess the protective immunity after a heterologous challenge. It should be noted that a single sublethal infection by *Shigella* does not protect against subsequent homologous or heterologous challenge (18). Indeed, we demonstrated that formulated L-DBF protected pre-exposed mice from heterologous lethal pulmonary challenges. Thus, formulated L-DBF has the potential to be a broadly protective vaccine against shigellosis in sporadic and endemic areas of the world, regardless of prior pathogen exposure and the host’s age.

## Materials and methods

### Materials

All reagents were from Sigma or Thermo Fisher, unless otherwise specified, and were chemical grade or higher. A. T. Maurelli from the University of Florida, Gainesville, FL supplied *S. flexneri* 2a 2457T. E. Barry from the University of Maryland, Baltimore, MD provided the *S. sonnei* 53G. *S. flexneri* 2457T and *S. sonnei* 53G were used as challenge strains in this study. These two strains are well characterized and extensively used because of their extensive genomic data and well-known pathogenic traits (29–31).

### Protein production and vaccine formulation production

The detailed procedure for producing IpaD, IpaB, and L-DBF has been thoroughly described previously (13). In summary, plasmids encoding IpaD, IpaB, or L-DBF were used to transform *E. coli* Tuner (DE3) cells. The transformed cells were then grown in a 10 L bioreactor. IPTG (1 mM) was used to induce protein expression after which the cells were allowed to grow for 3 h at 37°C. The bacteria were collected by centrifugation, resuspended in IMAC binding buffer (20 mM Tris-HCl pH 7.9, 500 mM NaCl, and 10 mM imidazole) with 0.1 mM AEBSF [(4-(2-aminoethyl) benzene sulfonyl fluoride hydrochloride], and lysed using a microfluidizer at 18,000 psi with three passes. After clarification by centrifugation, the supernatant was applied to a nickel-charged IMAC column. For the His-Tag IpaD (HT-IpaD), the protein was then eluted with IMAC elution buffer (20 mM Tris-HCl pH 8.0, 500 mM NaCl, and 500 mM imidazole), followed by a pass over a Q column to eliminate any contaminating LPS. After dialyzing against PBS, the protein was kept at -80°C.

The cognate IpaB chaperone HT-IpgC was co-expressed with IpaB and L-DBF. The IpaB/HT-IpgC and L-DBF/HT-IpgC complexes were purified using the IMAC protocol as described for HT-IpaD. Each complex was eluted with increasing imidazole and loaded onto a Q column. Lauryldimethylamine oxide (LDAO) was added to the pooled fractions at a final concentration of 0.1% to release L-DBF from the HT-IpgC chaperone. LDAO serves to solubilize membrane-associated proteins while preserving their biological activity and structural stability, which is needed for hydrophobic IpaB. The LDAO-treated complexes were passed over a second IMAC column with the IpaB and L-DBF present in the flow-through. These were dialyzed into PBS containing 0.05% LDAO and stored at -80°C. We have shown in our prior work that this concentration of LDAO, which is just above its critical micelle concentration, does not affect or hinder the host immune responses to L-DBF. EndoSafe cartridges and a NexGen PTS system (Charles River Laboratories, Wilmington, MA) was used to determine LPS levels. The Triton phase separation was conducted repeatedly, as needed, until the endotoxin level was below 5 endotoxin units per mg protein. All proteins had final endotoxin levels below 5 EU/mg protein, thereby adhering to the acceptable standards for preclinical animal research.

To produce an oil-in-water emulsion formulation, squalene (8% w/v) and polysorbate 80 (2% w/v weight) were mixed to achieve a homogenous oil phase. Polysorbate 80 was used as an emulsifying agent to stabilize the emulsion. Using a Silverson L5M-A standard high-speed mixer, 40 mM histidine (pH 6) and 20% sucrose were added to the oil phase with mixing at 7500 rpm, followed by six passes in a Microfluidics 110P microfluidizer at 20,000 psi to generate a milky emulsion of 4XME (MedImmune Emulsion) (32). To make the L-DBF with ME, the protein was added to the emulsion (diluted to 1XME) to a final concentration of 0.67 mg/ml, and incubated overnight at 4°C. The ME is an oil-in-water emulsion characterized by small droplet size (~120nm) that can be readily taken up and transported by dendritic cells to the lymph nodes (LN) and into germinal centers (GCs) to elicit a robust protective immune response (33). This oil-in-water emulsion adjuvant has been employed in vaccine research and has a proven safety and tolerability record in both clinical (34) and preclinical trials (35). It can be administered to mice without causing adverse effects.

### Mouse *S. flexneri/S. sonnei* pre-exposure, L-DBF vaccination, and challenge

**Figure 1:**
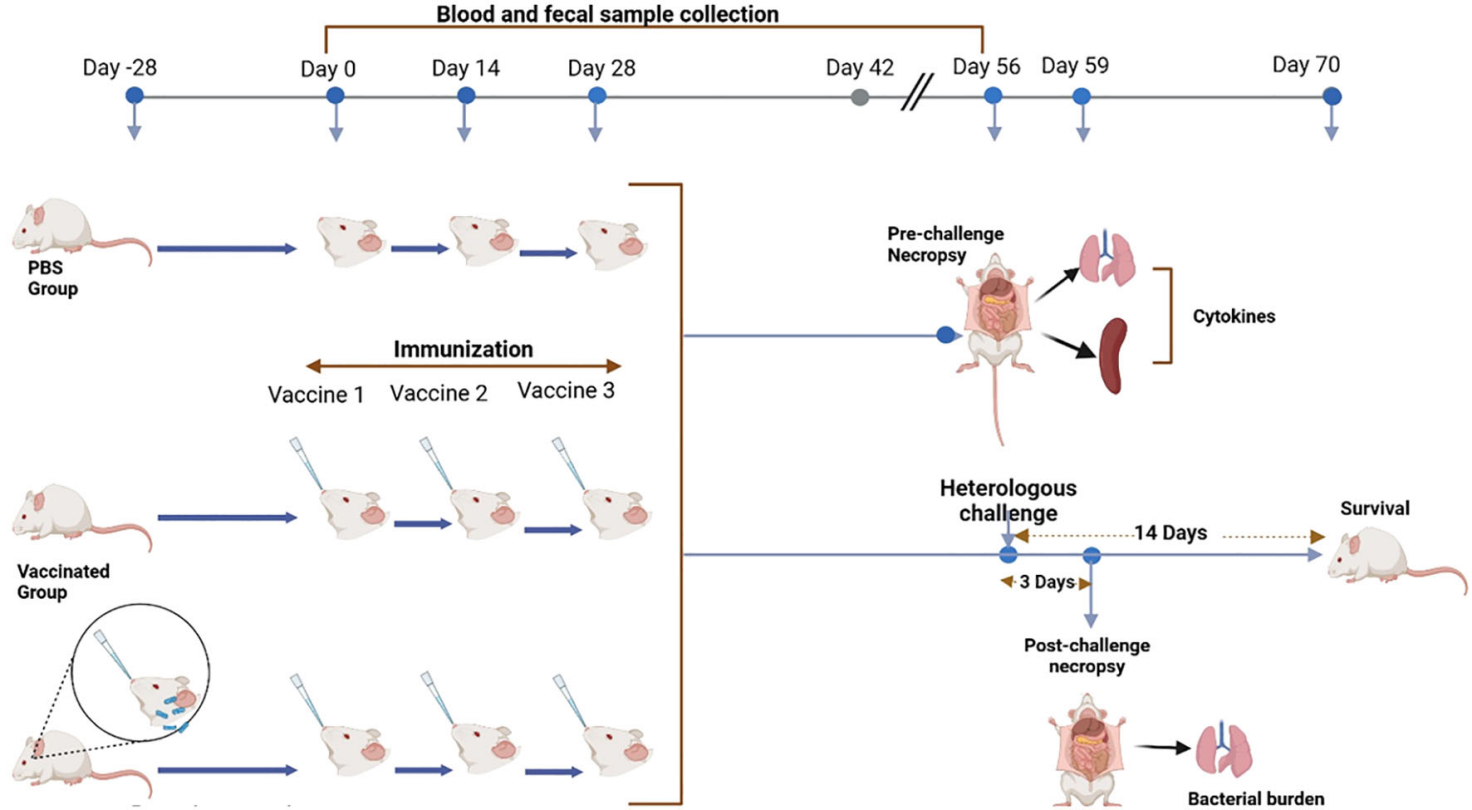
Summary of the experimental design showing all steps involved in the study. The arrows indicate the time points and progression of steps for the experimental procedures. (Figure generated using BioRender.com).

*S. sonnei* and *S. flexneri* were grown in tryptic soy agar (TSA) plates containing 0.025% Congo red overnight. Red colonies were selected for growth in tryptic soy broth (TSB) at 37°C with shaking at 200rpm until the absorbance (A_600_) reached 1.00. Bacteria were harvested by centrifugation and resuspended in PBS. For the single pre-exposure study, female 18 months-old BALB/c mice (n = 20/group, five for pre-challenge, five for post-challenge necropsy, ten for survival) were exposed/treated intranasally (IN) to PBS or 5 × 10^4^ CFU/25 µL *S. flexneri* 2a (SFV) or *S. sonnei* 53G (SSV) on day −28 (28 days before the first vaccination). All mice were weighed daily and the health score was monitored twice daily for 14 days after pre-exposure. The treated groups (SFV and SSV, respectively) were vaccinated IN with 5 µg L-DBF-ME in 30 µL per mouse on days 0, 14, and 28. Meanwhile, another group was vaccinated with L-DBF-ME without prior treatment with the pathogens (Vaccinated) and control groups were vaccinated with PBS. On day 56, 15 mice from each group were challenged with *S. flexneri* 2a or *S. sonnei* 53G (**Figure 1**). The remaining five mice were used to assess pre-challenge immune response at necropsy. The five challenged mice from each group were euthanized on day 3 post-challenge to assess immune response and to enumerate the bacterial burden in the different groups. The remaining ten mice in each group were monitored twice daily for weight loss and health scores for two weeks. Mice were euthanized if their weight loss exceeded 20% of their original weight for more than 72 hours (3 days), their blood glucose reached ≤100 mg/dL, or if they received a poor (moribund) health score. All animal protocols were reviewed and approved by the University of Missouri Institutional Animal Care and Use Committee Practices (Protocol number: 38241). Whenever possible, the number of animals was reduced, in particular where prior experiments already showed that a single pre-exposure to *S. flexneri* or *S. sonnei* did not provide protection from a homologous or heterologous lethal challenge (18).

### IgG BLISA and IgA ELISAs

Blood and fecal samples were collected on days 0, 14, 28, 42, and 56 for the determination of immunoglobulin titers as described previously with minor modifications (36). We used serum samples to determine anti-IpaD or anti-IpaB IgG titers by BLISA using the Octet^®^ system for biolayer interferometry using Ni-NTA (NTA) Biosensors (Sartorius, Göttingen, Germany). The experiments were performed using an Octet BLI discovery 13.0 software according to the manufacturer’s instructions. Briefly, Octet^®^ Ni-NTA (NTA) biosensors were washed with Tris-buffered saline with 0.05% Tween 20 (TBST) for 240 sec; charged with 10 mM nickel-sulfate for 60 sec; washed with TBST for 240 sec; and dipped into the wells containing either His-Tag IpaB or His-Tag IpaD (20 µg/ml in TBS) for 500 sec to load the antigen onto the sensors. The sensors were then washed with TBST for 240 sec; dipped in diluted serum samples for 500 sec to generate the Ag-Ab complex; and washed with TBST for 240 sec. The Ag-Ab coated sensors were then dipped into the solutions of the HRP-conjugated secondary antibodies (12.5 µg/ml in TBST) for 400 sec and washed with TBST for 240 sec. The IgG responses in the serum samples were analyzed in real time by the Octet BLI analysis studio 13.0 software. The 1mM EDTA was used to regenerate the sensors for future use. To measure the anti-IpaD or anti-IpaB IgA titers in fecal wash samples, microtiter plate wells were coated with 100 ng of either IpaB or IpaD in 100 µL PBS and incubated at 37°C for 2 h. Wells were incubated overnight in PBS using 10% nonfat dry milk. As the primary antibody source, duplicate fecal sample washes were added to the wells, and they were incubated for 1 h at 37°C. HRP-conjugated secondary antibodies (1:4000 dilution in PBST using 10% nonfat dry milk) were added after washing with PBS containing 0.05% Tween 20 and incubated for one h at 37°C. OPD substrate (o-phenylenediamine dihydrochloride) was added after a second wash and an ELISA plate reader was used to detect the signal at A_450_. By fitting antibody titrations to a five-parameter logistic model, endpoint titers were ascertained.

### Serum bactericidal assay

The serum bactericidal assay (SBA) was performed by using high-throughput imaging of the bacteria on filter plates as previously described (37). In summary, serum from each mouse group was combined to create the heat-inactivated pooled serum, which was then diluted two-fold in PBS in triplicate. A 96-well round bottom plate was used for the assay and 90 µL of the diluted serum and baby rabbit complement (Cedarlane, Burlington, NC) was added to each well of the plate. A freshly grown single red colony of *S. flexneri 2a* or *S. sonnei* 53G selected from a Congo Red TSA plate was sub-cultured in 10 mL of TSB at 37°C and 200 rpm of shaking. The culture was allowed to grow until the A_600_ reached 0.2. After adding *Shigella* serotypes (1 × 10^4^ CFU/10 µL) to each well, the plates were cultured at 130 rpm shaking for one h at 37°C. Twenty µL of each mix condition was added to wells of Millipore multiscreen HV filtration plates that had been ethanol-wetted with the liquid removed by vacuum. Every plate was sealed in a Ziploc bag and left to incubated overnight at 37°C with 5% CO_2_. Each well received 100 µL of a 0.01% solution of Coomassie blue R-250, which was swiftly evacuated by aspiration. Subsequently, 100 µL of a methanol-acetic acid destain solution was added to each well, which was shaken for 10 min at room temperature. Before counting, the plastic filter plate bottom was taken off and left to air dry after removing the destain solution by aspiration. A CTL immunospot reader (Cellular Technology Limited, Shaker Heights, OH) was used to count the CFUs. For the competitive SBA, 2 µg of IpaB, or IpaD in 45 µL PBS was added to the wells following two-fold dilutions in triplicate. A portion (90 µL) of a pooled mixture of serum from L-DBF immunized mice (1:16 for the IpaB or IpaD) was combined with baby rabbit complement in the appropriate wells. After gentle shaking at room temperature for 30 min, the *Shigella* sp of choice (1 × 10^4^ CFU/10 µL) were added to each well. The remaining steps were performed as above. The killing activity was measured by the following formula: killing % = (spots in control well – spots in test well)/spots in control well. The number of spots in control wells was statistically insignificantly different than the wells containing the complement and the bacteria. Thus, control wells were considered as a baseline and a negative control group.

### IFN-γ or IL-17A ELISpot assays

Splenocytes and lung cells from mice were isolated and homogenized as described previously (16). Using a FluoroSpot assay as per the manufacturer’s specifications (CTL), the cells were cultured for 24 h at 37°C in the presence of 5 µg/mL IpaB or IpaD in plates coated with antibodies against IFN-γ or IL-17 as directed by the manufacturer (CTL). The CTL Immunospot reader was utilized to quantify the cells that secreted cytokines.

### Cytokine determinations

Lung cells and splenocytes were cultured for 48 h at 37°C with 10 µg/mL IpaB, IpaD, or PBS in cell culture plates as described previously (16). Supernatants were collected and subjected to cytokine analysis using U-PLEX kits by the manufacturer’s instructions (Meso Scale Discovery, Rockville, MD). The concentrations of cytokines were measured with an MSD plate reader and related analytical software.

### Statistics

Statistical analysis was performed using the GraphPad Prism (version 10.1.1). Log-rank (Mantel-Cox) tests were used for survival tests. The ANOVA test was used for cytokine and SBA analysis. *P < 0.05; **P < 0.005; ***P < 0.0002, ****P < 0.0001.

## Results

### L-DBF protected mice in a lethal pulmonary challenge model by heterologous serotypes

**Figure 2:**
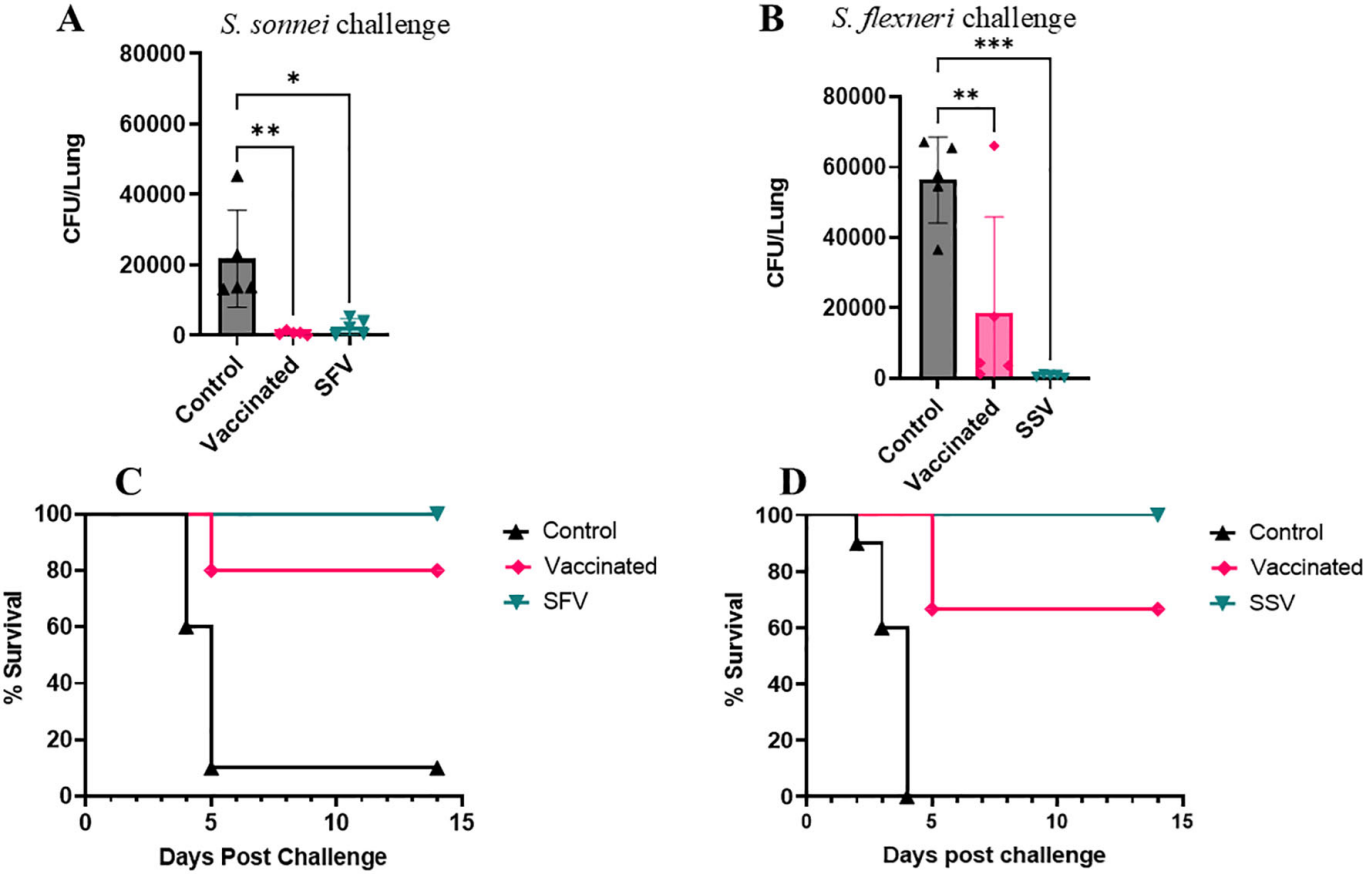
Protective efficacy of L-DBF against lethal *Shigella* challenge in mice. SFV and SSV groups were infected with sublethal doses of *Shigella* spp (**A, C**: *S. flexneri;* SFV or **B, D**: *S. sonnei;* SSV). All mice were vaccinated with PBS (control) or L-DBF/ME (Vaccinated, SFV and SSV groups) on days 0, 14, 28 and challenged four weeks after the last vaccination with *S. flexneri* (**B, D)** or *S. sonnei* **(A, C)** (1× 10^6^ CFU/mouse). Lung burden was measured in each group (n=5 mice/group) on day 3 post-challenge. The points represent individual CFU/lung values, and the SDs are denoted by error bars. Statistical analysis was done by two-way ANOVA (*P < 0.05; **P < 0.01; ***P < 0.001). The survival for each group (n=10 mice/group) was recorded for two weeks post-challenge.

The mice were challenged with a lethal pulmonary dose of the heterologous *S. sonnei* or *S. flexneri* on day 56 (28 days after the last immunization). Five mice per group were euthanized and the lung bacterial burden was enumerated on day 3 post-challenge. L-DBF/ME significantly reduced or prevented colonization of the *Shigella*, regardless of species, in vaccinated and pre-exposed vaccinated groups (SFV and SSV) compared to control mice (**Figures 2A, B**). Of the mice immunized with L-DBF/ME, 67% and 80% were protected from the lethal heterologous challenge by *S. flexneri* and *S. sonnei*, respectively, while 100% of mice in pre-exposure groups (SFV and SSV) were protected in both challenge studies (**Figures 2C, D**; **Table 1**). While a single prior sublethal infection is not sufficient to protect from a subsequent homologous or heterologous lethal challenge, these data may indicate that the prior infection can serve to prime the immune system for subsequent responses against IpaB and IpaD. Likewise, the body weight of surviving mice in pre-exposed vaccinated groups (SFV and SSV) recovered almost 100% of their original body weight at day 14 post-challenge and body weight in vaccinated groups gained more than 90% of their original body weight in both heterologous challenges (**Supplementary Figures S1A, B**).

**Table 1:** Protective efficacy of L-DBF/ME against *Shigella* spp.

Challenge Strains (CFU/mouse)	Attack Rate Vaccinated (ARV)		Attack Rate Unvaccinated (ARU)	Vaccine Efficacy (VE)	
	Vaccinated	SFV/SSV	Control	Vaccinated	SFV/SSV
*S. sonnei* 53G (1 x 10^6^)	20%	0	90%	70%	100%
*S. flexneri* 2a 2457T (1 x 10^6^)	33	0	100	67%	100%

Mice (n=10/group) were vaccinated with 5 μg L-DBF/ME (ARV) or PBS (ARU) and challenged with the indicated serotype at the stated dose. Vaccine Efficacy (VE) is shown where VE = 1–Attack Rate Vaccinated (ARV)/Attack Rate Unvaccinated (ARU).

### L-DBF induces IFN-γ and IL-17 secreting cells in the lungs

IFN-γ and IL-17 are crucial for the immune defense against *Shigella*, we focused our attention on these cytokines (38–40). The removal of intracellular bacteria relies on Th1 responses and the activation of macrophages, both of which are promoted by IFN-γ (41). To control pathogen infection at mucosal sites, it’s necessary to recruit neutrophils and produce antimicrobial peptides, which is assisted by IL-17, which plays a role in mucosal immunity (42). We prioritized IFN-γ and IL-17 due to their established importance and mechanistic role in *Shigella* infections, although other cytokines also contribute to immunity.

**Figure 3:**
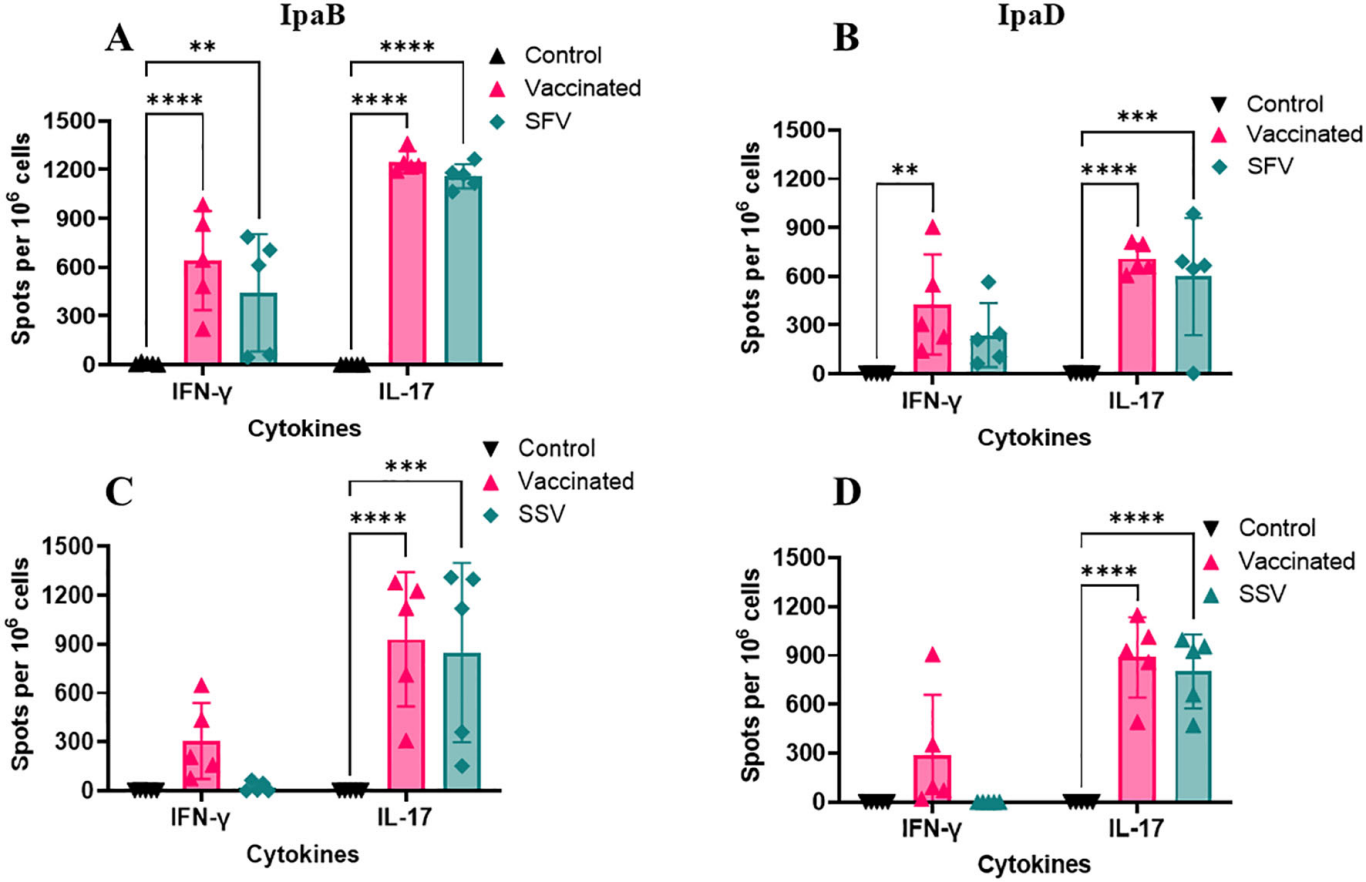
Frequency of IL-17 and IFN-γ secreting cells following antigen-specific stimulation. Single-cell lung suspensions were used to assess antigen-specific IFN-γ and IL-17 secreting cells using an ELISpot assay. Cells from each animal group [*S. flexneri* pre-exposure group A, B; and *S. sonnei* pre-exposure group C, D] were incubated either with 5 µg IpaB **(A, C)** or IpaD **(B, D)**. IFN-γ and IL-17 secreting cells were then enumerated by ELISpot as described in Materials and Methods and are presented here as spot-forming cells/10^6^ cells. The data are plotted as means ± SD for individual mice in each group. Significance was calculated by comparing the control group with L-DBF/ME vaccinated mice (vaccinated or SFV/SSV) using two-way ANOVA. (**P < 0.01; ***P < 0.001; ****P < 0.0001).

Lung cells were isolated from a subset of each mouse group prior to challenge and stimulated with either IpaB or IpaD to enumerate IFNγ- and IL-17 secreting cells using ELISpot. Regardless of pre-exposure condition, the lungs of the L-DBF/ME vaccinated mice showed higher frequencies of IL-17-secreting cells than those of the control mice (**Figure 3**). The systemic response of cytokine-secreting cells enumerated from isolated splenocytes by ELISpot analysis was relatively lower than lungs (**Supplementary Figure S2**). The IL-17-secreting cells in the spleen of vaccinated as well as SFV and SSV groups were higher than control groups for both IpaB and IpaD.

**Figure 4:**
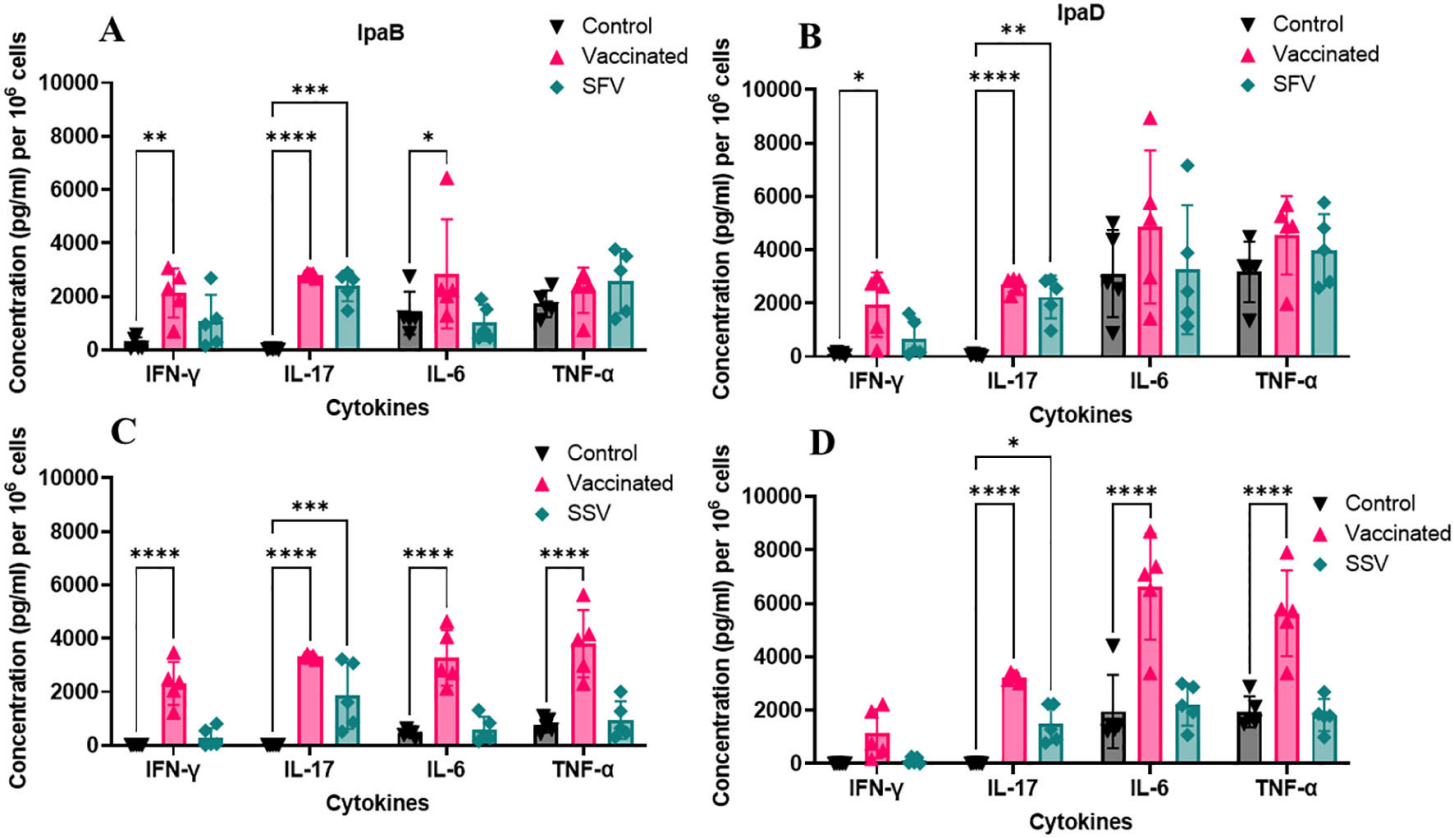
Quantification of cytokines secreted from lung cells. Data for lung cells from *S. flexneri* **(A, B)** and *S. sonnei* **(C, D)** pre-exposed mice following stimulation with IpaB **(A, C)** or IpaD **(B, D)** are presented. The cells were collected from the mice 28 days after the last vaccination. Cells were incubated with 10 µg IpaB or IpaD and cytokine levels were then measured by MesoScale Discovery analysis as per the manufacturer’s specifications. The data are presented here as pg/mL/10^6^ cells. The data are plotted as the mean ± SD for the individual mice in each group. Significance was calculated by comparing groups using two-way ANOVA. (*P < 0.05; **P < 0.01; ***P < 0.001, ****P < 0.0001).

To elicit a defense against mucosal infections, mucosal immunity must be stimulated and activated (43). Even though the number of cells secreting IFN-γ and IL-17 was considerably higher in the immunized animals, ELISpot does not quantify the total amount of cytokines released by these cells. We therefore stimulated lung cells and splenocytes with either IpaB or IpaD and measured cytokine levels secreted into the culture supernatant (**Figure 4**; **Supplementary Figure S3**, respectively). Lung cells from the vaccinated mice produced higher quantities of IFN-γ, IL-17A, IL-6, and TNF-α than those from the control groups, albeit not all were significantly higher (**Figure 4**). IL-17A, but not IFN-γ, IL-6, and TNF-α were secreted at significantly higher quantities by lung cells from pre-exposed groups (**Figure 4**). In the splenocytes, following stimulation with IpaB or IpaD, cells from vaccinated and SFV/SSV groups produced higher quantities of IL-17A than those from the control groups (**Supplementary Figure S3**). In addition, the SSV had a higher level of IFN-γ than those of control groups in splenocytes (**Supplementary Figures S3C, D**). The IL-17 responses from lung cells post-stimulation relied upon receiving the L-DBF/ME vaccine. The IL-17 responses from SFV/SSV groups were lower than the vaccinated group but still significantly higher than the control groups and helped protect mice from the lethal challenge. Though local IL-17 levels in lung cells in SFV and SSV seem lower than in the vaccinated group, their systemic response in spleen cells was similar or visibly higher.

### L-DBF-induced anti-IpaB and anti-IpaD antibodies in elderly mice

Because *Shigella* spp infection is widespread in endemic regions of the world, it is important to demonstrate that the elderly can develop protective immunity following L-DBF/ME vaccination regardless of prior exposure to *Shigella* spp. All mice vaccinated with L-DBF/ME elicited higher IgG and IgA responses regardless of prior exposure of the mice to *Shigella* serotypes (**Supplementary Figures S4, S5**). The anti-IpaD titers were lower than the anti-IpaB titers, which is consistent with the results from our previous investigations (16, 18). After the first immunization (as detected on day 14), SFV and SSV mice elicited higher fecal IgA and serum IgG titers than the vaccinated group (**Supplementary Figures S4, S5**), likely due to the vaccination at day 0 providing a boost after a prime resulting from the *Shigella* spp pre-exposure. All vaccinated mice showed maximal antibody titers, regardless of pre-exposure, after the first and second boost (as detected in days 28 and 42, respectively).

### L-DBF-induces antibodies are functional in serum bactericidal assays *in vitro*

**Figure 5:**
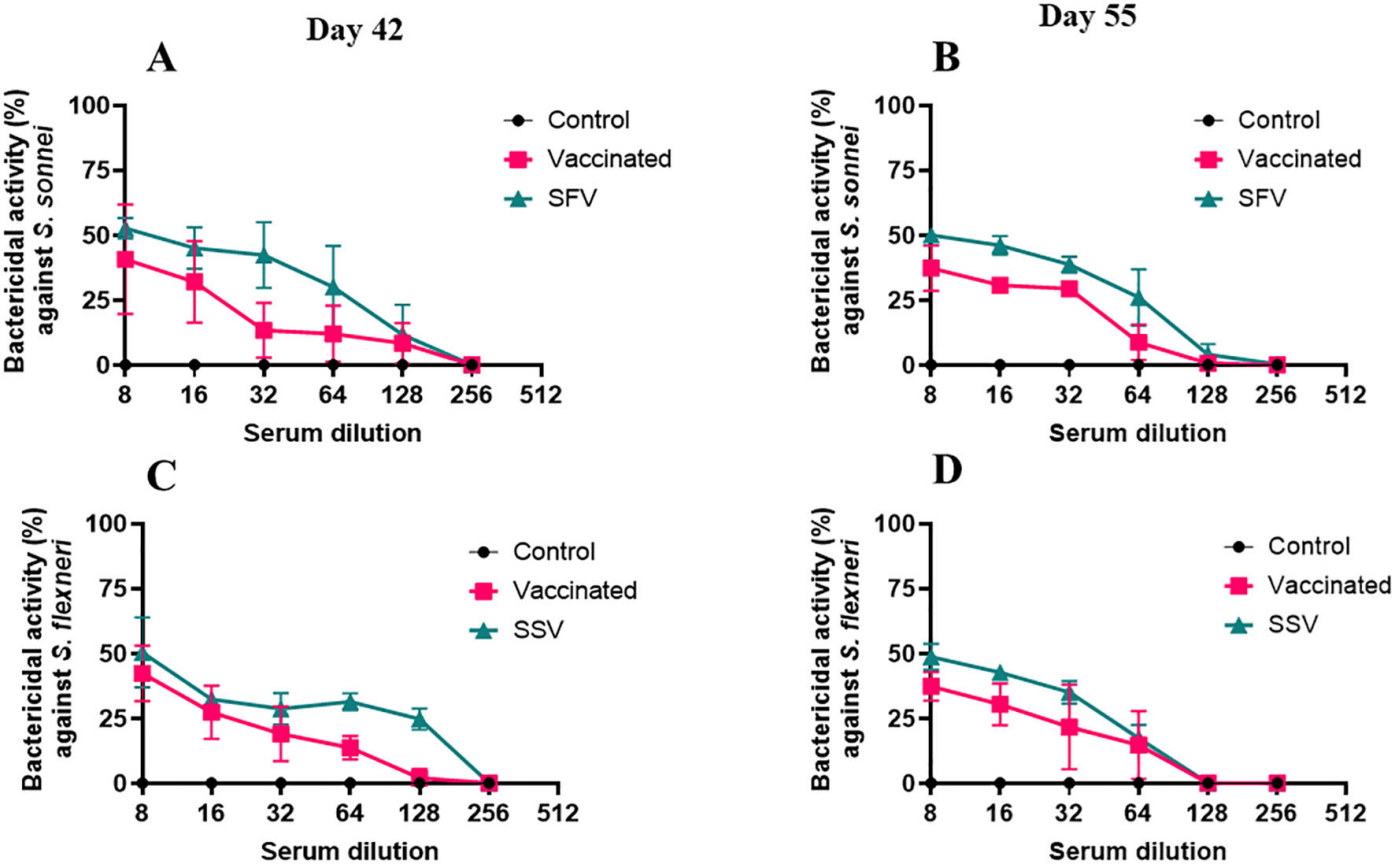
Serum bactericidal assay (SBA) in pool serum from mice of all groups. The SBA activity (%) in serially diluted serum collected on day 42 (**Figures 4A, C**) and 55 (**Figures 4B, D**) from vaccinated or SFV/SSV groups is shown. The heterologous serotype was used to test the SBA activity from *S. flexneri* treated mice **(A, B)** and *S. sonnei* treated mice **(C, D)**. CFU obtained from wells incubating with serum from the control groups were used as 0% SBA (baseline). SBA activity (%) = (spots in control well − spots in test well)/spots in control well. Triplicate wells for each test point are presented for each serum sample. The Mann-Whitney test was used for analysis (See **Supplementary Table 4** for the statistics of **Figure 4**). *p<0.05; **p<0.01; ***p< 0.001; ****p< 0.0001.

Serum bactericidal assay (SBA) measures the immunoglobulin’s ability to direct the killing of targeted bacteria via complement. Sera from both SFV and SSV and the naïve vaccinated groups possessed significant SBA activity while serum from control groups did not possess any SBA activity (0% as baseline). The serum from the vaccinated group collected on day 42 (two weeks after the last immunization) exhibited 40% (**Figure 5A**) and 42% (**Figure 5C**) bactericidal activity against *S. sonnei and S. flexneri*, respectively, at a 1:8 serum dilution. The bactericidal activity of the vaccinated group’s serum was statistically significant after a 1:64 serum dilution against *S. flexneri* (**Figure 5C**) and 1:16 dilution against *S. sonnei* (**Figure 5A**). In the case of day 55 serum collected from the vaccinated mice (28 days after the last immunization), the bactericidal activity was statistically significant against both *S. flexneri* (**Figure 5D**) and *S. sonnei* (**Figure 5B**) at a 1:64 dilution of the serum. However, serum collected on day 42 from the SFV group killed 52% of *S. sonnei* (**Figure 5A**), while the serum from the SSV group killed 50% of *S. flexneri* (**Figure 5C**) at a 1:8 serum dilution. This bactericidal activity was significantly higher than the control group up to a 1:64 dilution for SFV serum against *S. sonnei* (**Figure 5A**) and to a 1:128 dilution for SSV serum against *S. flexneri* (**Figure 5C**). The sera collected on day 55 from the SFV and SSV groups killed 50% of *S. sonnei* (**Figure 5B**) and 48% *of S. flexneri* (**Figure 5D**), respectively. When comparing the day 42 and day 55 sera samples, the vaccinated group dropped to 0% bactericidal activity against *S. sonnei* after a 1:256 dilution (**Figure 5A**) and a 1:128 serum dilution against *S. flexneri* (**Figure 5B**). In the case of day 55 sera, the bactericidal activity dropped to 0% after a 1:128 serum dilution against both serotypes (**Figures 5B, D**). The bactericidal activity of serum samples from treated groups dropped at 0% after 1:256 serum dilution on day 42 sample (**Figures 5A, C**) and 1:128 serum dilution for the day 55 sample (**Figures 5B, D**). Next, using IpaB and IpaD as competitors for the bactericidal function, we evaluated the bactericidal activity of the pooled sera from all groups (**Supplementary Figures S6, S7**). Since the addition of IpaB blocked the bactericidal activity in a concentration-dependent manner, the SBA for the vaccinated, SFV, and SSV mice primarily targeted IpaB rather than IpaD, determined using the heterologous serotypes. The use of heterologous strains ensures that the observed SBA activity is not due to anti-LPS antibodies. On days 42 or 55, the bactericidal activity for vaccinated pooled serum with 2 µg or 1 µg IpaB added was noticeably less than that for those without IpaB added (**Supplementary Figure S6**). When IpaD was added, this was not the case (**Supplementary Figure S7**). These findings imply that whereas SBA exhibited bactericidal action in both the pre-exposed and L-DBF/ME vaccinated mice groups, this activity may differ based on the immunogenicity of the antigen used in the immunization of the animals.

## Discussion

Both naïve and pre-exposed mice immunized with L-DBF/ME generated antigen-specific serum IgG and fecal IgA in this study. Previously, we demonstrated 100% protection against different serotypes of *Shigella* for L-DBF immunized mice in an adult mouse pulmonary model (16). In this study, L-DBF/ME protected 70 and 67% of the naïve elderly mice against *S. sonnei* and *S. flexneri* lethal challenge, respectively. The reduced protection in elderly mice compared to adults might be due to immunosenescence (44), however, 100% of elderly mice exposed to *Shigella* spp previously were protected by L-DBF/ME from the lethal heterologous challenge in this study. Thus, previous infection has the potential to augment protective efficacy of L-DBF/ME immunization by providing a prime exposure to IpaB and IpaD without exhibiting any detrimental consequences.

We have previously shown that, in adult mice, L-DBF elicits both a local lung response and a systemic humoral response to effectively mount a protective anti-*Shigella* response (16, 18). The detection of serum IgG against IpaB and IpaD of *Shigella* suggests the activation of a systemic humoral response in the vaccinated mice. Meanwhile, the presence of mucosal IgA detected in fecal samples against these antigens reflects a parallel activation of the mucosal humoral immune response. Moreover, synthesis of IgA demonstrates the involvement and activation of intestinal immunological network, which is a fundamental feature of the systemic mucosal immune system (45). Mucosal bacterial pathogens like *Shigella* possess a variety of pathogenic strategies including the ability to alter host immunological and inflammatory responses, and to control host cell death and survival signaling pathways. These characteristics make it possible for pathogens to take advantage of cellular and immunological responses, adapt to the environment of the intestinal mucosa, and initiate infection (46). Thus, a successful vaccine should be able to activate mucosal immunity to elicit protection against infection by mucosal pathogens (43).

The L-DBF/ME induced fecal IgA synthesis in all immunized groups regardless of prior exposure to the pathogen in the elderly mice used in this study. The overall IgA synthesis in elderly mice was comparatively lower than that of adult mice in our previous report (18), which can be attributed to the reduced antigen presentation capacity of dendritic cells in elderly mice as well as low B cell, memory cell, and plasma cell counts compared to young and adult mice (44, 47). Furthermore, compared to younger people, older people’s vaccine-specific antibodies are substantially less functional due to reduced class switching (48). Age-related declines in the production of vaccine-specific IgA have been associated with this loss of function (49, 50). L-DBF/ME immunized mice produced IpaB-specific IgG titers in all immunized groups regardless of pre-exposure to the *Shigella* serotypes in this study. The *S. sonnei* pre-exposed group had higher IgG responses compared to L-DBF-vaccinated mice. *S. sonnei* also has prolonged survival ability within the host due to its extracellular lifestyle capability (51) and the type VI secretion system (T6SS) that *S. sonnei* encodes gives it a gut competitive edge and longer persistence than *S. flexneri* (52). This could potentially allow more time for the immune system to mount a stronger antibody response. Anti-IpaB-specific IgG was reported to be more important in clinical protection than anti-IpaD antibodies (53). Generally, antibodies from older individuals are less functional than adults at neutralizing and opsonizing pathogens during natural infection (54). Anti-IpaB antibodies produced by L-DBF immunization in elderly mice in the present study had complement-mediated *in vitro* bactericidal activity regardless of prior exposure of the host to the *Shigella* serotypes, which promoted pathogen clearance in what would otherwise be a lethal infection. All the pre-exposed mice (100%) in the current study were protected against heterologous challenge, even though protection from natural infection by *Shigella* serotypes only shields hosts from future infection by homologous serotypes and even that requires multiple pre-exposure events (55). This additional protection is believed to be due to the sublethal infection serving as a prime for the L-DBF and increasing the levels of IpaB and IpaD antibodies following L-DBF vaccination.

To get the best protection from a natural infection and to improve the effectiveness of the vaccine, adequate memory T cell responses must complement the humoral immune responses. The development of the anti-*Shigella* immunity appears to be supported by the induction of a protective T cell response in the form of Th1/Th2 and Th17 cells in this study, with an emphasis on Th17 cells. The widely recognized cytokine milieu hypothesis postulates that the cytokine milieu determines the Th subset of effector T cells that arise upon CD4 T cell activation by the antigen. This protection depends on the presence of specific cytokines that have a type-specific response. In this instance, Th1 is required in response to IFN-γ, whereas IL-17A indicates Th17 cell involvement. The protection that our immunization platform provides in elderly mice seems to be associated mostly with the Th17 response. It is crucial to have this cytokine for protection against intracellular infections. Through developing tertiary immunity in lymphoid tissues, IL-17 can help in preventing bacterial invasion (56, 57). Although lung inflammation is typically caused by IL-17 downstream signaling in mice receiving PBS vaccinations, the Th17 pathway in mice that have received vaccinations generally is linked to the host’s defense regardless of prior pathogen exposure and the age of the host.

With the emergence of multidrug-resistant bacteria, most antibiotics, including fluoroquinolones and extended-spectrum cephalosporins, are no longer fully effective against shigellosis (58). The World Health Organization (WHO) listed *Shigella* to the “high” priority group on its list of antimicrobial-resistant organisms that represent a serious concern to public health due to their growing resistance to antibiotics. Multidrug-resistant *Shigella* serotypes are challenging to treat and this has made them the main cause of *Shigella* infections in endemic regions of the world (59). In our previous work using the mouse model, we found that two exposures to a single *Shigella* serotype can shield the host from a later homologous infection, but a single exposure is not sufficient for this. Nevertheless, a host cannot be protected from heterologous serotypes even by repeated exposure to a single distinct serotype (18). Therefore, it is essential to create an effective vaccination that can shield a host with a history of shigellosis from *Shigella* infection. From the data presented here, we suggest that hosts with a history of prior *Shigella* infection should be protected from a heterologous infection by a *Shigella* L-DBF vaccination. Thus, L-DBF is a strong vaccine candidate for the endemic regions of the world where people are exposed to multiple *Shigella* serotypes throughout their lives.

## Data Availability

The original contributions presented in the study are included in the article/**Supplementary Material**. Further inquiries can be directed to the corresponding author.
