# Supplementary material for: The fusion subunit vaccine L-DBF protects aged mice against heterologous lethal Shigella challenge after prior exposure
Source: Front Immunol. 2025 Jun 5;16:1586537. doi: 10.3389/fimmu.2025.1586537 (PMC12176820; doi:10.3389/fimmu.2025.1586537)
Supplement: Supplementary file 1 [file DataSheet1.docx]

**Supplemental Information for:**

**The fusion subunit vaccine L-DBF protects aged mice against heterologous lethal *Shigella* challenge after prior exposure**

Md Shafiullah Parvej, Ti Lu, Suhrid Maiti, Zackary K. Dietz, Debaki R. Howlader, Mst Nusrat Zahan, Alexa Cato, Satabdi Biswas, William D. Picking, Wendy L. Picking^*^

Bond Life Sciences Center and the Department of Veterinary Pathobiology, University of Missouri, Columbia, Missouri, USA

*Corresponding author: E-mail: [wendy.picking@missouri.edu](mailto:wendy.picking@missouri.edu). (WLP)

**Key words:** *Shigella*, subunit vaccine, aged mice, intranasal vaccination, pre-exposure

**Supplemental Table S1.  Acronyms used in this paper.**

MDR Multi-drug resistance

IMAC Immobilized metal affinity chromatography

WHO World Health Organization

T3SS Type III secretion system

LMIC Low- or Middle-Income Country

T-V Treatment-Vaccine

TSA Trypticase soy agar

TSB Tryptic soy broth

TBST Tris-buffered saline with Tween® 20

SFV *Shigella flexneri* treatment-Vaccine

SSV *Shigella sonnei* treatment-Vaccine

ELISA Enzyme-Linked Immunosorbent Assay

BLI Biolayer interferometry

DBF IpaD-IpaB fusion protein

dmLT Double-mutant heat-labile enterotoxin

LTA1 A1 moiety of the active subunit of dmLT

L-DBF LTA1 fusion with DBF

LPS Lipopolysaccharide

LDAO Lauryl dimethylamine oxide

ETEC Enterotoxigenic *Escherichia coli*

IN Intranasal

ME MedImmune emulsion

IPTG Isopropyl β-D-1-thiogalactopyranoside

PBS Phosphate buffered Saline


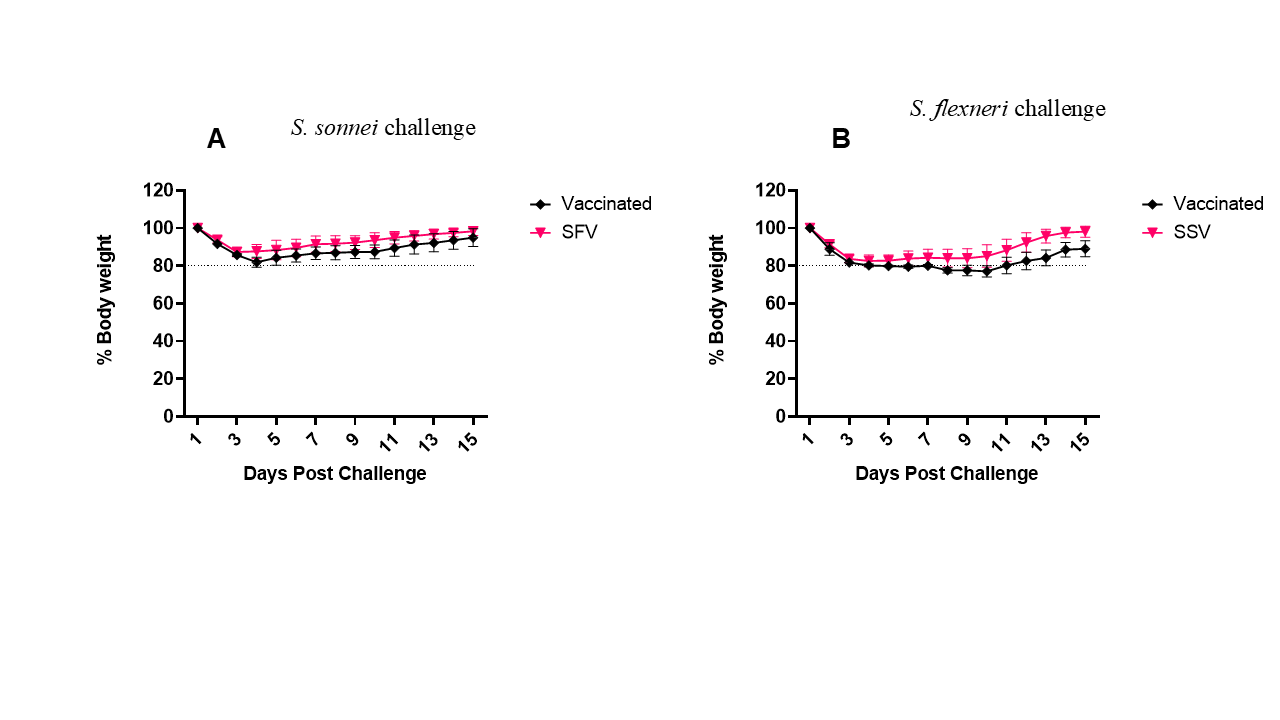


**Supplemental Figure S1:** The body weights were recorded for mice after lethal *Shigella* challenge. Balb/C mice (n=10) were vaccinated intranasally (IN) on days 0, 14 and 28 with PBS or 5 μg L-DBF/ME. On day 56, the mice were challenged IN with 1×10^6^ CFU/mouse of *S. sonnei* 53G (A) or *S. flexneri* (B). A single preexposure with a sublethal dose of *S. flexneri* (A) or *S. sonnei* (B) (5×10^4^ CFU/mouse) was given to SFV and SSV groups 28 days before the first immunization, respectively. Mice were monitored twice daily for health scores and once daily for body weight.


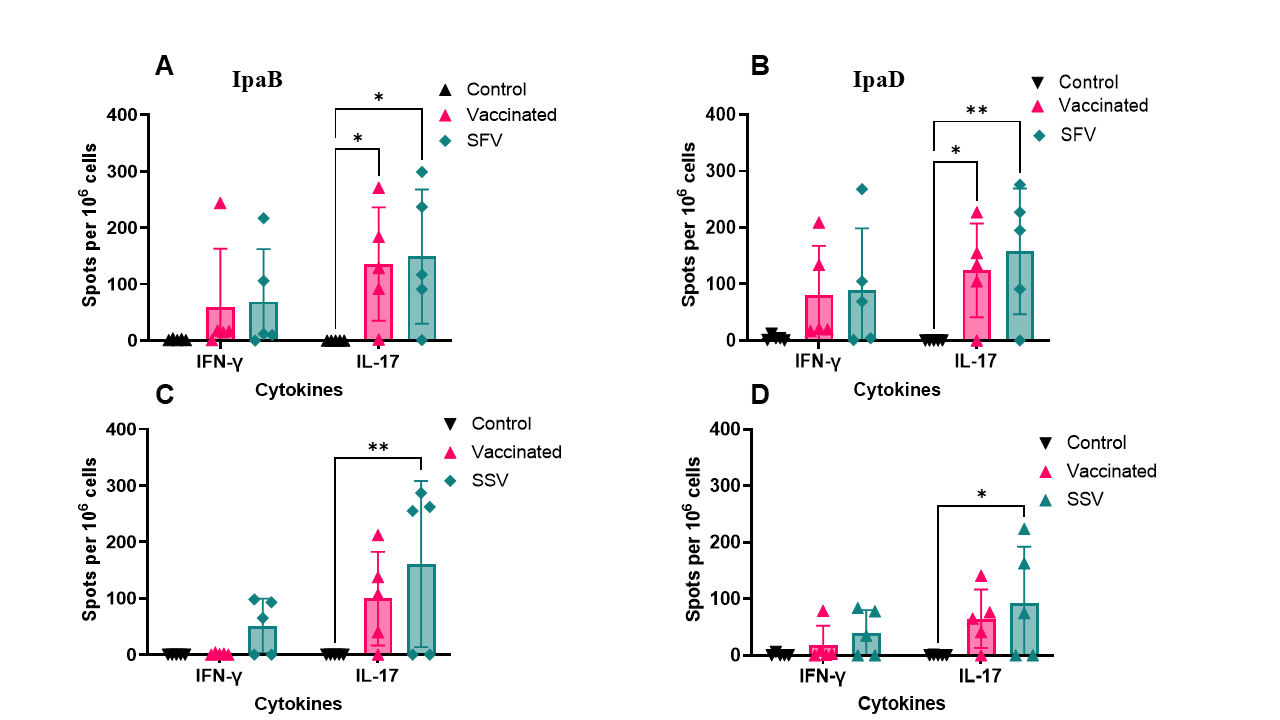


**Supplemental Figure S2: Quantification of cytokines secreted cells from splenocytes.** Data are shown for *S. flexneri* pre-exposure experiments (A, B) and *S. sonnei* preexposure experiment (C, D) after stimulation with either IpaB (A, C) or IpaD (B, D). Splenocytes were collected from mice at 28 days after the last vaccination. Cells were incubated with either 10 µg IpaB or IpaD. IFN-γ and IL-17 secreting cells were then enumerated by ELISpot as described in Materials and Methods. They are presented here as spot-forming cells/10^6^ cells. The data are plotted as means ± SD for individual mice in each group. Using two-way ANOVA, significance was calculated by comparing unvaccinated groups (PBS) and mice vaccinated with L-DBF/ME. (*P < 0.05; **P < 0.01)


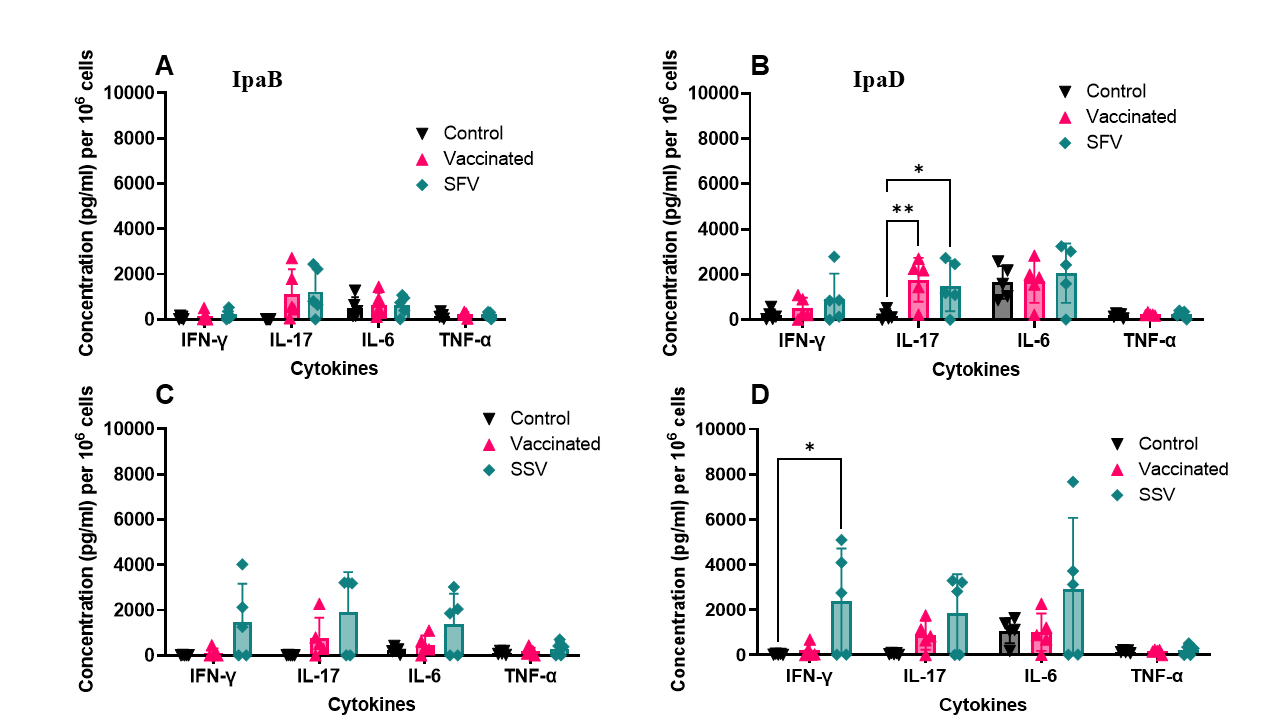


**Supplemental Figure S3: Quantification of cytokines secreted from splenocytes.** Data are shown for *S. flexneri* pre-exposure (A, B) and *S. sonnei* pre-exposure (C, D) after stimulation with IpaB (A, C) or IpaD (B, D). Splenocytes were collected from the mice 28 days after the last vaccination. Cells were incubated with 10 µg IpaB or IpaD. Cytokine levels were then measured by Meso Scale Discovery analysis as per the manufacturer’s specifications and are presented here as pg/mL/10^6^ cells. The data are plotted as the mean ± SD for the individual mice in each group. Significance was calculated by comparing groups using two-way ANOVA (*P < 0.05; **P < 0.01). Cells from both the SFV and SSV groups produced higher quantities of IL-17A than those from the control groups. In A and B, IpaB stimulation for control versus vaccinated mice is P=0.14; control versus SFV is P=0.10; IpaD stimulation for control versus vaccinated and control versus SFV were statistically significant (P≤0.05). In C and D, IpaB stimulation for: control versus vaccinated is P=0.20; control versus SSV is P=0.11; IpaD stimulation for control versus vaccinated is P= 0.49 and control versus SSV is P=0.05). In addition, the SSV had a higher level of IFN-γ than was seen for control groups in splenocytes (Fig C and D, IpaB stimulation for: control versus SSV is P= 0.19; IpaD stimulation for control versus SSV is P= 0.01).


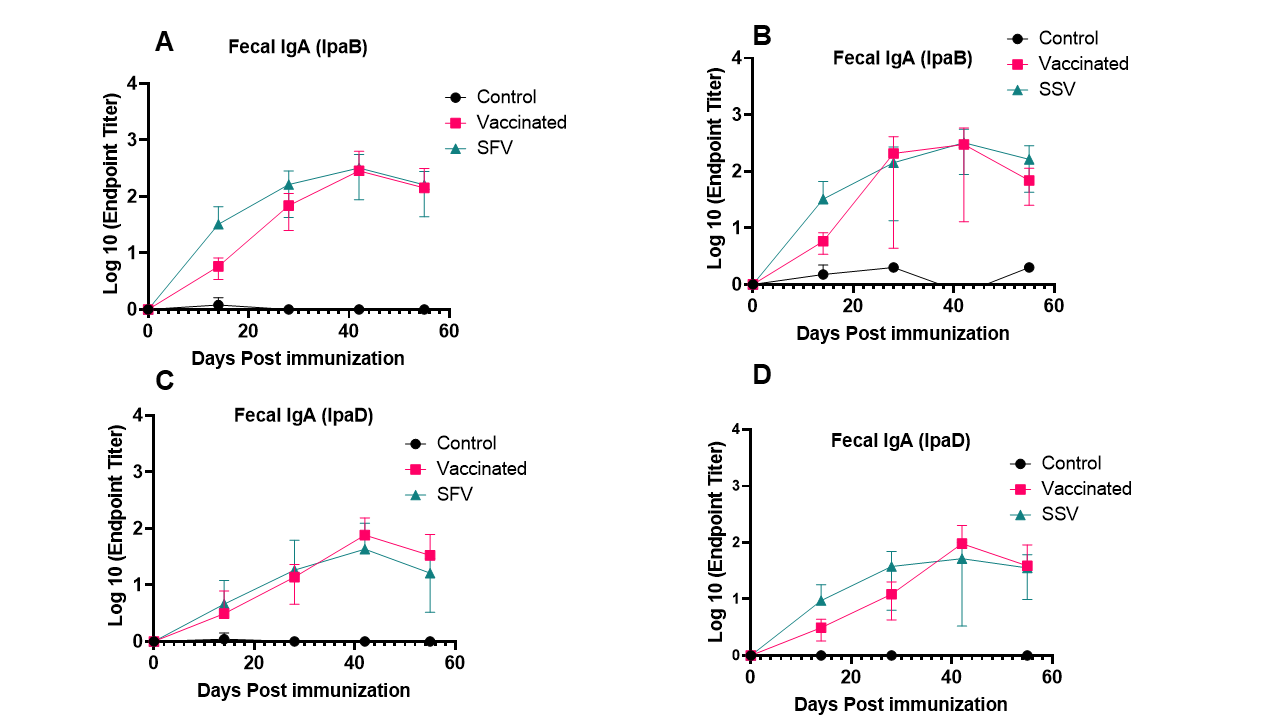


**Supplemental Figure S4: Kinetics of fecal IgA titers detected by ELISA.**  The SFV (A, C) and SSV serum IgG titers are presented (B, D).  SFV, SSV and vaccinated mice were vaccinated IN three times (days 0, 14, and 28). Fecal samples were collected and titers for anti-IpaB and anti-IpaD IgA titers by ELISA. Each point represents the mean of each group (n = 10/group).


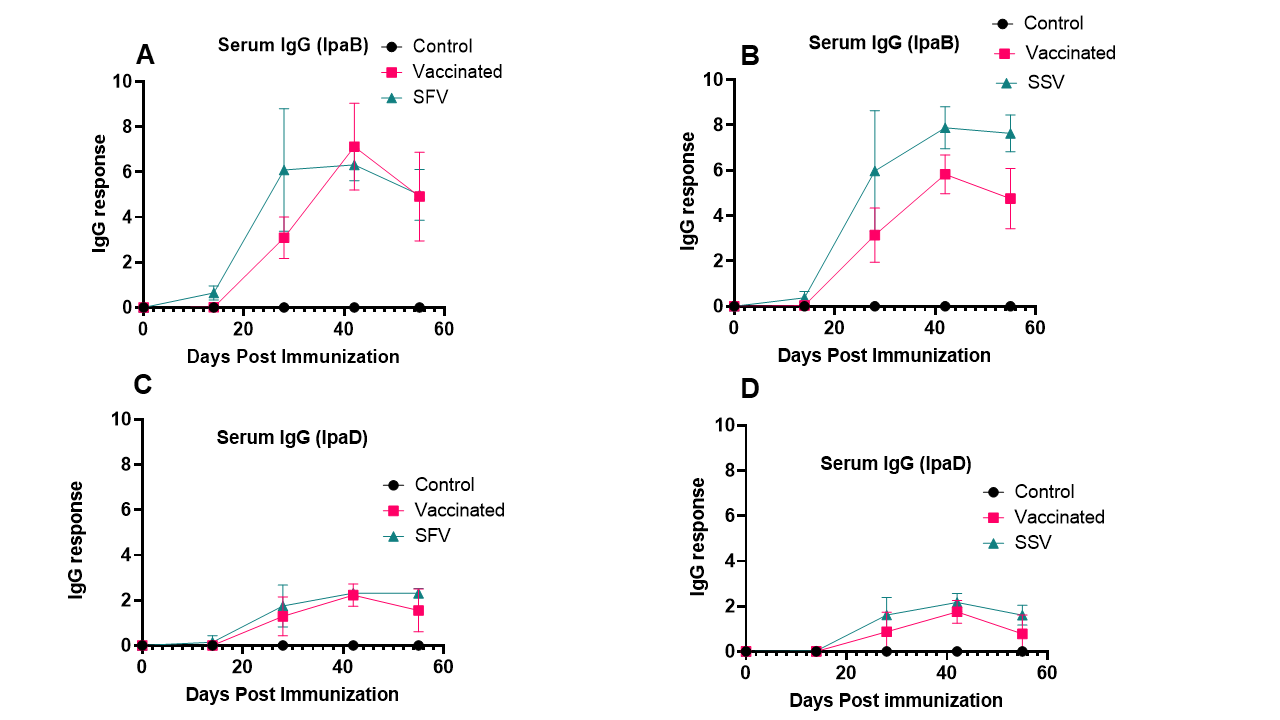


**Supplemental Figure S5: Kinetics of Serum IgG titers detected by BLISA**. The SFV (A, C) and SSV (B, D) serum IgG titers are presented.  SFV, SSV and vaccinated mice were vaccinated IN three times (days 0, 14, and 28). Serum samples were collected and titers for anti-IpaB and anti-IpaD IgG response were by BLISA. Each point represents the mean of each group (n = 7/group).


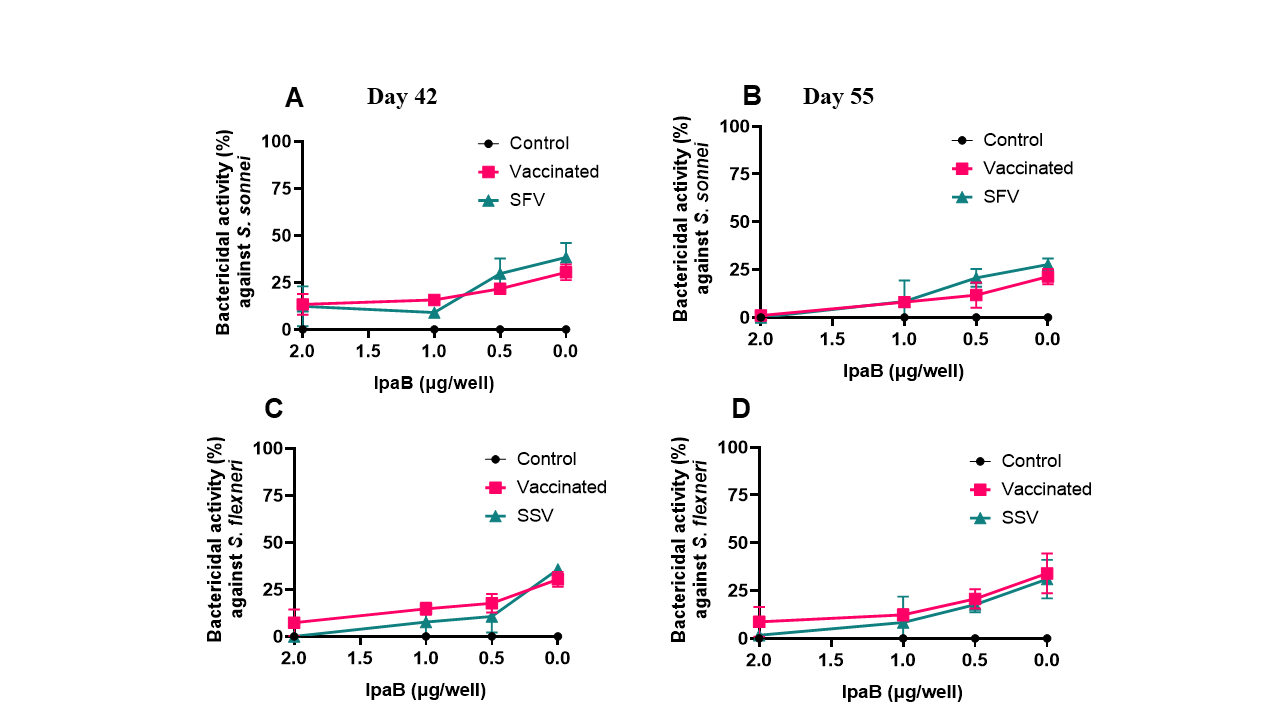


**Supplemental Figure S6:** SBA for serum from mouse groups in competition with exogenously added IpaB. The bactericidal activity (%) for serum obtained from SFV and SSV groups (1:16) and NT-V groups (1:16) in competition with different concentrations of IpaB on days 42 (A, C) or 55 (B, D) is shown. Heterologous serotypes were used to determine the bactericidal activity in SFV (A, B) and SSV (C, D). The 0% bactericidal activity baseline was the CFU counts from wells incubated with serum from the control groups. Spots in control well minus spots in test well)/spots in control well were used to calculate the bactericidal activity. For every test point, experiments were conducted in triplicate wells. * Mann-Whitney test. *p<0.05; **p<0.01; ***p< 0.001; ****p< 0.0001


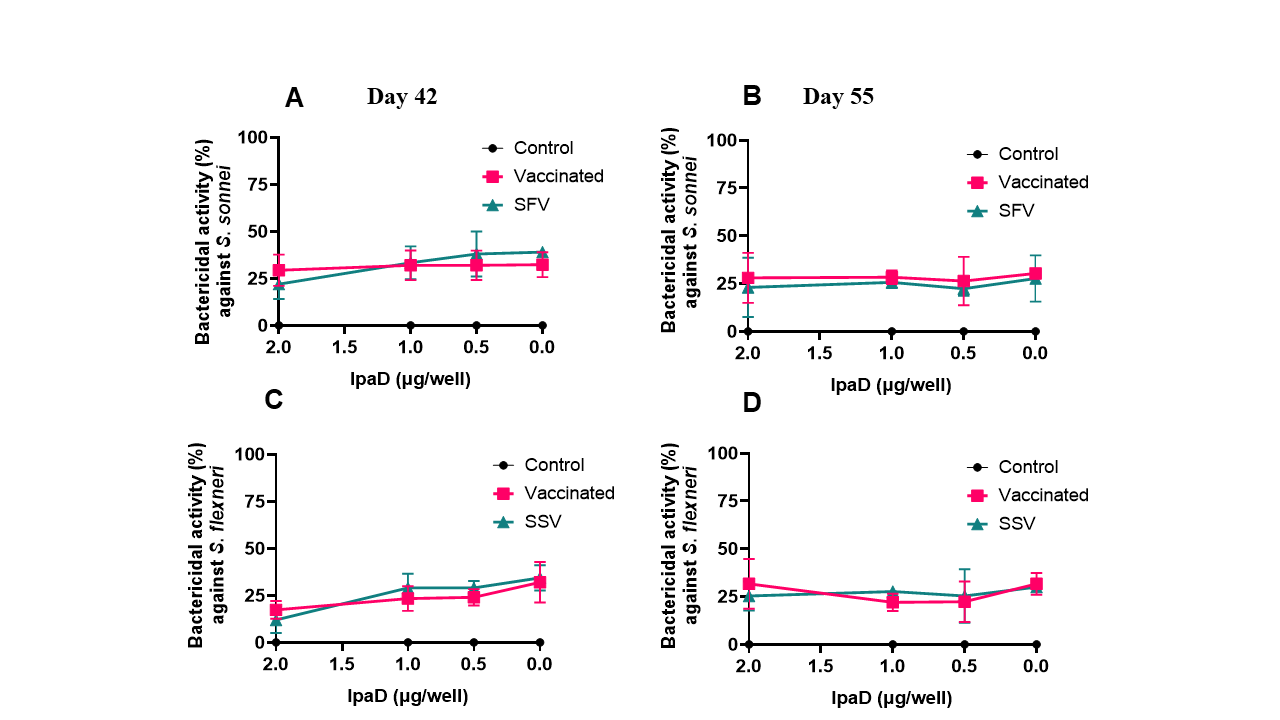


**Supplemental Figure S7:** SBA for serum from mouse groups in competition with exogenously added IpaD. The bactericidal activity (%) in serum obtained from SFV and SSV groups (1:16) and NT-V groups (1:16) in competition with different concentrations of IpaD on days 42 (A, C) or 55 (B, D) is shown. Heterologous serotypes were used to determine the bactericidal activity in SFV (A, B) and SSV (C, D). The 0% bactericidal baseline was the CFU counts from wells incubated with serum from the control groups. Spots in control well minus spots in test well)/spots in control well were used to calculate the bactericidal activity. For every test point, experiments were conducted in triplicate wells. * Mann-Whitney test. *p<0.05; **p<0.01; ***p< 0.001; ****p< 0.0001

**Supplemental Table S2: Statistics for the data shown in Figure 5A**

| Dilutions | P Value | | |
| --- | --- | --- | --- |
|  | Control VS. Vaccinated | Control VS. SFV | Vaccinated VS. SFV |
| 8 | <0.0001 **** | <0.0001 **** | 0.2774 ns |
| 16 | 0.0006 *** | <0.0001 **** | 0.2243 ns |
| 32 | 0.2083 ns | <0.0001 **** | 0.0017 ** |
| 64 | 0.2774 ns | 0.0012 ** | 0.0637 ns |
| 128 | 0.5319 ns | 0.2968 ns | 0.9024 ns |
| 256 | >0.9999 ns | >0.9999 ns | >0.9999 ns |

*p<0.05; **p<0.01; ***p< 0.001; ****p< 0.0001

**Supplemental Table S3: Statistics for the data shown in Figure 5B**

| Dilutions | P Value | | |
| --- | --- | --- | --- |
|  | Control VS. Vaccinated | Control VS SFV | Vaccinated VS SFV |
| 8 | <0.0001 **** | <0.0001 **** | 0.0014 ** |
| 16 | <0.0001 **** | <0.0001 **** | 0.0001 *** |
| 32 | <0.0001 **** | <0.0001 **** | 0.0201 * |
| 64 | 0.0327* | <0.0001 **** | <0.0001 **** |
| 128 | 0.9777 ns | 0.4530 ns | 0.5746 ns |
| 256 | >0.9999 ns | >0.9999 ns | >0.9999 ns |

*p<0.05; **p<0.01; ***p< 0.001; ****p< 0.0001

**Supplemental Table S4: Statistics for the data shown in Figure 5C**

| Dilutions | P Value | | |
| --- | --- | --- | --- |
|  | Control VS. Vaccinated | Control VS SSV | Vaccinated VS SSV |
| 8 | <0.0001 **** | <0.0001 **** | 0.2263 ns |
| 16 | <0.0001 **** | <0.0001 **** | 0.5505 ns |
| 32 | 0.0009 *** | <0.0001 **** | 0.1193 ns |
| 64 | 0.0182 * | <0.0001 **** | 0.0020 ** |
| 128 | 0.9075 ns | <0.0001 **** | <0.0001 **** |
| 256 | >0.9999 ns | >0.9999 ns | >0.9999 ns |

*p<0.05; **p<0.01; ***p< 0.001; ****p< 0.0001

**Supplemental Table S5: Statistics for the data shown in Figure 5D**

| Dilutions | P Value | | |
| --- | --- | --- | --- |
|  | Control VS. Vaccinated | Control VS. SSV | Vaccinated VS. SSV |
| 8 | <0.0001 **** | <0.0001 **** | 0.0552 ns |
| 16 | <0.0001 **** | <0.0001 **** | 0.0341 * |
| 32 | 0.0002 *** | <0.0001 **** | 0.0205* |
| 64 | 0.0101 * | 0.0022 ** | 0.8397 ns |
| 128 | >0.9999 ns | >0.9999 ns | >0.9999 ns |
| 256 | >0.9999 ns | >0.9999 ns | >0.9999 ns |

*p<0.05; **p<0.01; ***p< 0.001; ****p< 0.0001

**Supplemental Table S6: Statistics for the data shown in Supplemental Figure 6A**

| Dilutions | P Value | | |
| --- | --- | --- | --- |
|  | Control VS. Vaccinated | Control VS. SFV | Vaccinated VS. SFV |
| 2 | 0.9561 ns | >0.9999 ns | 0.9561 ns |
| 1 | 0.0774 ns | 0.0638 ns | 0.9950 ns |
| 0.5 | 0.0076 ** | <0.0001 **** | 0.0428 * |
| 0 | <0.0001 **** | <0.0001 **** | 0.1881 ns |

*p<0.05; **p<0.01; ***p< 0.001; ****p< 0.0001

**Supplemental Table S7: Statistics for the data shown in Supplemental Figure 6B**

| Dilutions | P Value | | |
| --- | --- | --- | --- |
|  | Control VS. Vaccinated | Control VS. SFV | Vaccinated VS. SFV |
| 2 | 0.0080 ** | 0.0143 * | 0.9667 ns |
| 1 | 0.0020 ** | 0.0858 ns | 0.2432 ns |
| 0.5 | <0.0001 **** | <0.0001 **** | 0.1376 ns |
| 0 | <0.0001 **** | <0.0001 **** | 0.1376 ns |

*p<0.05; **p<0.01; ***p< 0.001; ****p< 0.0001

**Supplemental Table S8: Statistics for the data shown in Supplemental Figure 6C**

| Dilutions | P Value | | |
| --- | --- | --- | --- |
|  | Control VS. Vaccinated | Control VS. SSV | Vaccinated VS. SSV |
| 2 | 0.1515 ns | >0.9999 ns | 0.1515 ns |
| 1 | 0.0021 ** | 0.1291 ns | 0.1770 ns |
| 0.5 | 0.0003 *** | 0.0253 * | 0.1770 ns |
| 0 | <0.0001 **** | <0.0001 **** | 0.3542 ns |

*p<0.05; **p<0.01; ***p< 0.001; ****p< 0.0001

**Supplemental Table S9: Statistics for the data shown in Supplemental Figure 6D**

| Dilutions | P Value | | |
| --- | --- | --- | --- |
|  | Control VS. Vaccinated | Control VS. SSV | Vaccinated VS. SSV |
| 2 | 0.2573 ns | 0.9481 ns | 0.4049 ns |
| 1 | 0.0745 ns | 0.2835 ns | 0.7383 ns |
| 0.5 | 0.0021 ** | 0.0082 ** | 0.8422 ns |
| 0 | <0.0001 **** | <0.0001 **** | 0.8422 ns |

*p<0.05; **p<0.01; ***p< 0.001; ****p< 0.0001

**Supplemental Table S10: Statistics for the data shown in Supplemental Figure 7A**

| Dilutions | P Value | | |
| --- | --- | --- | --- |
|  | Control VS. Vaccinated | Control VS. SFV | Vaccinated VS. SFV |
| 2 | <0.0001 **** | 0.0012** | 0.3776 ns |
| 1 | <0.0001 **** | <0.0001 **** | 0.9669 ns |
| 0.5 | <0.0001 **** | <0.0001 **** | 0.5161 ns |
| 0 | <0.0001 **** | <0.0001 **** | 0.4444 ns |

*p<0.05; **p<0.01; ***p< 0.001; ****p< 0.0001

**Supplemental Table S11: Statistics for the data shown in Supplemental Figure 7B**

| Dilutions | P Value | | |
| --- | --- | --- | --- |
|  | Control VS. Vaccinated | Control VS. SFV | Vaccinated VS. SFV |
| 2 | 0.0007 *** | 0.0047 ** | 0.7258 ns |
| 1 | 0.0006 *** | 0.0017 ** | 0.9120 ns |
| 0.5 | 0.0013** | 0.0060 ** | 0.8137 ns |
| 0 | 0.0003 *** | 0.0008 *** | 0.9120 ns |

*p<0.05; **p<0.01; ***p< 0.001; ****p< 0.0001

**Supplemental Table S12: Statistics for the data shown in Supplemental Figure 7C**

| Dilutions | P Value | | |
| --- | --- | --- | --- |
|  | Control VS. Vaccinated | Control VS. SSV | Vaccinated VS. SSV |
| 2 | 0.0021 ** | 0.0346 * | 0.4727 ns |
| 1 | <0.0001 **** | <0.0001 **** | 0.4307 ns |
| 0.5 | <0.0001 **** | <0.0001 **** | 0.5162 ns |
| 0 | <0.0001 **** | <0.0001 **** | 0.8629 ns |

*p<0.05; **p<0.01; ***p< 0.001; ****p< 0.0001

**Supplemental Table S13: Statistics for the data shown in Supplemental Figure 7D**

| Dilutions | P Value | | |
| --- | --- | --- | --- |
|  | Control VS. Vaccinated | Control VS. SSV | Vaccinated VS. SSV |
| 2 | <0.0001 **** | 0.0005 *** | 0.5218 ns |
| 1 | 0.0023 ** | 0.0002 *** | 0.5923 ns |
| 0.5 | 0.0019 ** | 0.0005 *** | 0.8613 ns |
| 0 | <0.0001 **** | <0.0001 **** | 0.9547 ns |

*p<0.05; **p<0.01; ***p< 0.001; ****p< 0.0001
